# Evaluation of drought-tolerant chickpea genotypes (*Cicer arietinum* L.) using morphophysiological and phytochemical traits

**DOI:** 10.3389/fpls.2025.1529177

**Published:** 2025-04-09

**Authors:** Bahman Fazeli-Nasab, Saeedreza Vessal, Abdolreza Bagheri, Saeid Malekzadeh-Shafaroudi

**Affiliations:** Department of Biotechnology and Plant Breeding, Faculty of Agriculture, Ferdowsi University of Mashhad, Mashhad, Iran

**Keywords:** drought-stress, proline, malondialdehyde, root depth, root length

## Abstract

Chickpea (*Cicer arietinum* L.), through a series of morphological, physiological, biochemical, and molecular changes, would tolerate abiotic stresses such as water deficiency. Accordingly, two separate experiments were conducted to investigate phytochemical and morphophysiological traits of various candidate chickpea genotypes in response to drought stress. In the first experiment, morphological and phytochemical traits were evaluated by maintaining pots at 70% water holding capacity (WHC) and applying gradual drought stress (to 50% and 25% WHC) to four- to six-week-old seedlings. In the second experiment, the stressed plants were exposed to progressive drought stress for biochemical measurements, while control plants were irrigated at 70% WHC. The highest photosynthetic water use efficiency (9.94 µmolCo_2_/µmolH_2_o) under drought stress belonged to the MCC552 genotype, followed by the MCC696 genotype with 7.25. The highest chlorophyll content (SCMR) was recorded in MCC537 (0.99 µg/cm²), followed by MCC352 (0.89 µg/cm²). The deepest root depth (70.83 cm) was observed in MCC537, followed by MCC552 (69.36 cm). Root diameter increase under stress conditions compared to normal conditions only in MCC352 and MCC552. However, leaf area was higher in MCC552 and MCC537 under drought stress conditions. The SCMR(μg/cm²) was highest in the MCC552 (1.48), followed by MCC696(1.32) and MCC80 (1.31). The highest proline level was observed in the MCC552, which increased with drought stress severity. The lowest level of Malondialdehyde was observed in the MCC696 genotypes, while the highest catalase level was found in the MCC696, followed by the MCC537 and MCC552. Based on root depth, root length, diameter, leaf area, as well as phytochemicals traits, especially proline, MCC552 and MCC696 were identified as the most tolerant genotypes to drought stress.

## Introduction

The pulses family has 18000 species and 650 genera (Varshney et al., 2009, Varshney et al., 2013). The cultivated chickpea, *Cicer arietinum* L., belongs to the genus Cicer, which includes ten annual and 36 perennial species. Of all the yearly species, *C. arietinum* L. is the only one domesticated and cultivated globally (Toker et al., 2021; Kakaei et al., 2024). Chickpea is the second most crucial legume crop worldwide, with 59 chickpea-producing countries. It has a global cultivation area of 18.86 million hectares, a production of 15.08 million tons, and an average yield of 1.01 tons per hectare in 2020 (FaoSAT Rome, 2023). It is mainly produced in marginal lands under adverse conditions (biotic and abiotic stresses) with an average yield of about one ton per hectare, much less than its yield potential (6 tons per hectare under optimal conditions). A combination of drought, heat, cold, and salinity stresses negatively affects the productivity of chickpeas (Roorkiwal et al., 2020; Arriagada et al., 2022). In Iran, despite its relatively high cultivation area (660,000 hectares), its yield is much lower than the global yield average (495 kg.ha^-1^) (FaoSAT Rome, 2023).

Chickpea is primarily grown in arid and semi-arid environments, specifically on low-quality lands. These regions are characterized by challenging abiotic conditions, including extreme temperatures and drought stress. These environmental factors significantly limit chickpea production at different stages of growth throughout the growing season. Drought stress and high temperatures are two of the most critical challenges, resulting in a decrease in yield of up to 50% and 20% globally, respectively (Sachdeva et al., 2018). In this scenario, it is essential to identify and develop productive chickpea genotypes using traditional and modern breeding methods. The goal is to create new varieties of chickpeas that are resilient to climate change and can thrive in various environments. This is crucial to food security in the short and long term (Arriagada et al., 2022).

Despite the ability of chickpeas to grow under stress conditions, there is still a need to increase the tolerance characteristics against drought stress by improving the efficiency of the plant in water deficit conditions (Keerthi et al., 2023) through a better specific root architecture (Sinha et al., 2016). Due to the direct contact of the roots with soil particles, they act as the first signal transducer of drought stress (Agrawal et al., 2016).

Plant growth hinges on the intricate balance between roots and shoots, shaping overall development. Among environmental challenges, water scarcity poses the greatest threat to plant vitality. Drought stress triggers a wide range of responses, from cellular changes to altered growth patterns. To fully grasp plant drought resistance, it’s essential to examine both visible morphological adaptations and internal biochemical shifts. This comprehensive approach unveils the complex strategies plants employ to endure water-limited conditions, providing crucial insights into their resilience mechanisms and potential strategies for enhancing crop performance in challenging environments (Kang et al., 2022; Kou et al., 2022).

Drought stress gradually diminishes the absorption of CO2 as it impairs stomatal function. It also decreases leaf area, shoot expansion, and root proliferation, interrupting the plant’s water relations. Moreover, it disrupts photosynthetic pigments and hampers gas exchange, reducing plant growth and productivity (Anjum et al., 2011; Quagliata et al., 2023; Wang et al., 2023).

The absence of water lowers the water potential of the soil, leading to a decrease in the number of leaves per plant, the size of each leaf (leaf area), and the lifespan of the leaves. The reduction in leaf area due to drought stress is caused by a decrease in the rate of photosynthesis (Anjum et al., 2011; Quagliata et al., 2023; Wang et al., 2023). The growth of plants is often limited by the rate of photosynthesis, especially when there is reduced soil water availability. This is because a decrease in chlorophyll content leads to a change in leaf color from green to yellow, indicating a reduction in the photosystem II reaction center. As a result, the absorption of photosynthetically active rays (PAR) is reduced, affecting water consumption (Lonbani and Arzani, 2011). A common adverse effect of drought stress on crops is decreased fresh and dry biomass production (Wang et al., 2023).

In the breeding program, it is essential to consider identifying physiological traits that contribute to drought tolerance. This is because separate genetic loci control seed yield and drought resistance. Therefore, utilizing physiological characteristics as an indirect selection is crucial in improving yield-based selection methods. Selection efficiency can be improved if specific physiological and morphological characteristics related to yield are identified under a stress environment and used as a selection criterion (Lonbani and Arzani, 2011; Khadka et al., 2020; Sewore et al., 2023). A variety of traits have been proposed to enhance selection efficiency and serve as indirect criteria for improving yield under stress conditions. These include stomata features (size, number, and conductance), leaf traits (area, shape, expansion, aging, maturity, cuticular resistance), root attributes (length and dry weight), water use efficiency (WUE), relative water content (RWC), evapotranspiration efficiency, and levels of phytochemical materials and physiological metabolites such as gayacol peroxidase (GPX), ascorbate peroxidase (APX), catalase (CAT), malondialdehyde (MDA), abscisic acid. as well as the stability of cell membranes. In addition, traits related to drought resistance, such as plant height, reduced leaf area, and early maturity, contribute to total seasonal evapotranspiration. Drought-tolerant cultivars usually maintain higher leaf RWC under drought stress (Lonbani and Arzani, 2011; Hashmi et al., 2023; Sewore et al., 2023).

To effectively implement any strategy for genetic improvement, it is crucial to utilize genetic diversity within the species for specific traits. Additionally, it is essential to develop breeding methods based on Mendelian genetic principles to ensure efficient selection processes (Acquaah, 2015). Adopting new breeding technologies is expected to enhance chickpea crop productivity greatly. Although conventional breeding methods have already yielded improved chickpea cultivars, there is still ample opportunity for further productivity gains. By integrating modern genomic resources with traditional breeding approaches, it is anticipated that chickpea varieties with resistance to both biological and climatic challenges can be introduced within a relatively short timeframe (Roorkiwal et al., 2020).

To evaluate drought-tolerant chickpea genotypes (*C. arietinum* L.) using morphological, physiological, and phytochemical traits, researchers have conducted various studies to select high-yielding genotypes for drought tolerance. The evaluation process involves assessing physio-biochemical indices, multi-environment yield performance, and different seedling traits. Chickpea genotypes have varied significantly in seedling traits and physio-chemical attributes under various environments. Physio-biochemical traits like proline, glycine betaine, and RWC have been reflected in chickpeas’ capability to tolerate drought stress (Shah et al., 2020; Arif et al., 2021; Hussain et al., 2021).

A significant obstacle in chickpea breeding programs is the limited genetic diversity. This poses a challenge in developing new and improved chickpea varieties that can withstand various abiotic stresses in the long term (Jha, 2018). To enhance the efficiency of breeding efforts, it is crucial to expand the genetic lines of chickpeas (Raina et al., 2019). In this context, the selection of new lines should rely on a thorough phenotypic and genetic description of the plants utilized as parental plants in breeding programs. This process can be accomplished using advanced phenotyping techniques (Mir et al., 2019), such as root system architecture in drought stress experiments (Brunel-Saldias et al., 2020). Our original project aims to evaluate the morphophysiological and transcriptomic analysis of candidate chickpea genotypes using the RNAseq in response to drought stress. Still, in this research, six drought-tolerant candidate genotypes, previously identified as superior in various field experiments were evaluated based on phytochemical and morphophysiological traits related to shoot and root systems, serving as additional criteria for effective selection under drought stress. One drought- tolerant genotype will be selected and compared with a sensitive genotype, such as ILC3279 to determine resistant genes to drought of chickpeas.

## Materials and methods

Over the past decade, extensive studies have been conducted to select and identify Kabuli-type chickpea genotypes with drought tolerance. Initial experiments focused on evaluating yield under drought stress in field conditions. Subsequent selection processes in greenhouse and laboratory settings revealed significant variation in drought-related traits among Kabuli-type chickpea genotypes. The finding from these studies (Ganjeali and Kafi, 2007; Amiri Deh Ahmadi et al., 2010; Ganjeali and Bagheri, 2010; Palta et al., 2010; Ganjeali et al., 2011b, Ganjeali et al., 2011c; Rahbarian et al., 2011; Raheleh et al., 2012) have led to the identification and acquisition of a list of candidate drought-tolerant genotypes (**Table 1**) used in this study.

**Table 1:** Characteristics of used chickpea genotypes.

Name^*^	Abbreviation name	Type	Origin	Ref
MCC552	552	Kabuli	Iran, native accession	(Ganjeali et al., 2011c)
MCC352	352	Kabuli	ICARDA (12247)	(Ganjeali et al., 2011a)
MCC427	427	Kabuli	Iran, Bojnurd native accession	(Ganjeali et al., 2011a)
MCC80	80	Kabuli	ICARDA (5311)	(Ganjeali et al., 2011c)
MCC696	969	Kabuli	Iran	(Abrishamchi et al., 2012)
MCC537	537	Kabuli	Iran, Gonabad native accession	(Ganjeali et al., 2011c)

MCC, Mashhad Chickpea Collection. *, Significant at the 0.05 (p < 0.05) level.

### Cultivation and drought stress improvement

The experiment was divided into two parts to measure morphological and phytochemical traits. Before sowing the seeds in the relevant experiment, they were disinfected with sodium hypochlorite (1%) and washed thrice with sterile distilled water.

### Experiment 1: root characteristics and morphological traits

**Figure 1:**
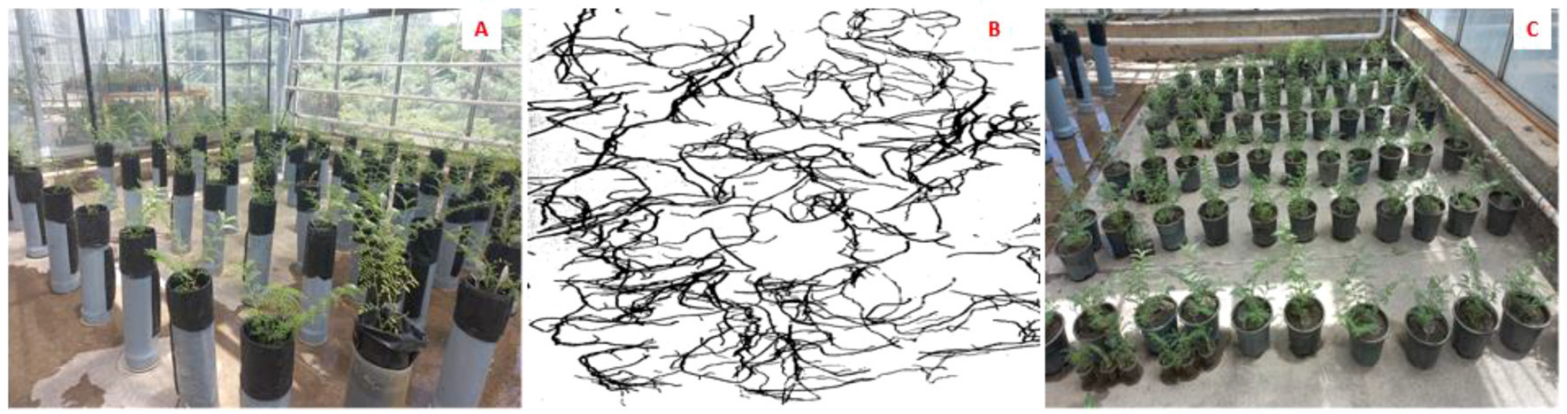
Five weeks old chickpea seedlings in three-liter pots for biochemical measurements under drought stress **(A)**; Chickpea seedling in 80 cm high PVC tube cylinders for morphological measurements, as well as root depth and length two weeks after exposure to drought stress **(B)** and a representative, scanned root profile **(C)**.

A cylindrical cultivation test (**Figure 1A**) was designed and implemented in controlled greenhouse conditions to evaluate the tolerant chickpea candidate genotypes using morphological traits and rooting status. This experiment used 80 cm high PVC tube cylinders for deep root growth. The soil texture consisted of 50% agricultural soil, 45% sand, and 5% Vermicompost. These soil textures were mixed and stirred repeatedly until the mixture was uniform. The sand and soil particles used were homogenized using a sieve. The cylinders were filled to 90 cm high and 35cm diameter. It used the same weight (8 kg) and height (80 cm) of soil texture in all PVCs to keep the soil compact.

Three seeds per cylinder were cultivated with three replications for the control and drought stress treatments in the greenhouse with a temperature of 25 ± 3°C and a humidity level of 50-70%. Only one seedling was kept in each cylinder one week after seedling emergence. Four weeks after emergence, chickpea plants were exposed to drought stress for one week at the 50% level of WHC and then gradually to severe drought stress of 25% WHC for another week based on our previous findings for creating progressive drought stress (Vessal et al., 2011, Vessal et al., 2012). In contrast, control plants were irrigated normally to keep 70% WHC during this time.

Two weeks after the application of drought stress, the plant shoots were cut off from the soil surface (from the root crown) and used to measure morphological traits. To extract the roots from the soil, the soil was gently washed with water, and it was tried not to cause any damage to the roots. The roots were separately washed and extracted. To measure the root profile, the average diameter and total length (including main tap root, secondary, and tertiary roots) of the roots along each cylinder were divided into two parts (35 cm up and 35 cm down in each cylinder). Root area, diameter, and length were measured and scanned [using a scanner (Image Analysis - Delta-T Devices) and HP Precision Scan Pro software] (**Figure 1B**). To analyze root properties (root length, root diameter and root surface area) (supplementary file (**Supplementary Figure S1**)), Delta-T Scan was used. Roots were scanned to a black and white images at a resolution of 400 dpi, and HP precisionScan Pro Software was also utilized.

The root depth was calculated from the distance from the root crown to the tip of the root [supplementary file (**Supplementary Figure S1**)]. All genotypes were evaluated in terms of morphological traits including root diameter, root density, root dry weight to total plant dry weight ratio, shoot dry weight, plant height, mesophyll conductivity, photosynthetic rate, photosynthetic water use efficiency (PWUE) (Sajid et al., 2023), transmission rate, EC, leaf area (it was measured by leaf area meter (Li-1300) device and WinDIAS2.0 software), stems number, MSI(Membrane stability index) (Sajid et al., 2023), stem diameter, chlorophyll (SCMR((SPAD chlorophyll meter reading)), leaf water potential and RWC (Li et al., 2023). Physiological parameters: The net photosynthe-sis rate, transpiration rate and stomatal conductance of fully expanded and well light exposed leaves were measured with a portable porometer (LCI4, ADC BioScientific Ltd. Hoddesdon, Herts, England). The JMicroVisionv1.2 software was used to record the leaf area after imaging accurately. To measure the leaf area the leaf area of five plants was immediately measured after sampling using a leaf surface measurement device. The mean value was then calculated to estimate the leaf area index (LAI), the total leaf surface area to the ground surface unit. Net photosynthesis, transpiration, stomatal conductance, and substomatal CO2 concentration were measured using an infrared gas analyzer (IRGA, Model LCA4, ADC BioScientific Ltd., Herts, UK). The photosynthetic pigment content of the plants was measured at the beginning of the flowering stage (35 days after planting; DAP). This was done using a handheld SPAD chlorophyll meter (Minolta 502) on the second youngest fully developed leaf from the top of each plant. Leaf chlorophyll fluorescence was measured using a fluorometer (Model OS1 FL, Opti-Sciences Inc., Hudson, USA) under light conditions. The youngest fully expended leaf was placed inside the chamber. The size of the leaf chamber of LCA4 is 6.25 cm-2. In this chamber, the air flows continuously, and the amount of light is adjusted using LED lamps. The time to measure photosynthesis was between 10 and 12AM.

### Experiment 2: biochemical measurements

Three seeds were shown in each three-liter pot (**Figure 1C**) with three replications for both control and drought stress treatments under greenhouse conditions with the same temperature and humidity in experiment 1. One week after emergence, only two seedlings were kept in each pot for applying stress treatment. After four weeks of growing under normal irrigation (70% WHC), seedlings were exposed to progressive drought stress by complete water withholding. Leaf samples from fully expanded leaves at the top of the plant were obtained at three-time points after water withholding (0, 48 and 96 hours) to analyzed biochemical traits (it is processed by microtiter plate reader BioTek and Gen5 software(BioTek Instruments, Winooski, VT, USA)) including proline, CAT, guaiacol peroxidase (GPX), APX, and MDA (Tuberosa and Salvi, 2024; Martin et al., 2013; Çevik et al., 2019). A factorial experiment was conducted using a completely randomized design (CRD) with three replications. Experiment factors were stress levels (0, 48, and 96 hrs after WHC) and the genotypes.

### Preparation of phytochemical extract

To prepare a 50ml extraction buffer, 0.607g of Tris and 0.05g of PVP were dissolved in 40ml of distilled water. The pH was adjusted to 8 using chloric acid, and the volume was brought to 50ml. For protein and phytochemical extraction, 0.5g of leaf sample was ground into powder using liquid nitrogen. 2ml of the prepared buffer was added to the ground sample and homogenized. The mixture was then centrifuged at 13,000 rpm for 15 minutes. The resulting supernatant was separated for measuring protein content and phytochemical activity. This method provided an efficient way to extract and prepare leaf samples for further biochemical analysis (Bradford, 1976).

### Enzymatic activity of guaiacol peroxidase

The activity of guaiacol peroxidase was measured using guaiacol as a substrate. A reaction mixture contained 25µL of phytochemical materials extract of the plant, 2.77mL of potassium phosphate buffer (50 mM and with pH= 7), 100 µL of 1% H_2_O_2,_ and 100 µL of 4% guaiacol. The increase in absorbance due to guaiacol oxidation was measured at 470 nm wavelength in 3 minutes. Each unit of enzyme concentration is defined as the amount of phytochemical materials that cause a 0.01 change in absorbance (Zhang et al., 2005). The concentration of enzyme was calculated using the extinction coefficient (ϵ = 26.6 mmol^-1^cm^-1^) using the following formula;


A=ϵbc

A: read absorbance equivalent,

b: Cuvette length,

c: the concentration of the H_2_O_2_

The concentration of enzyme was expressed as fresh weight units per gram (Nakano and Asada, 1981).

### Ascorbate peroxidase activity

APX activity was measured using a spectrophotometric method based on ascorbate oxidation. The reaction mixture contained 50 mM potassium phosphate buffer with an acidity of 7, 0.5 mM ascorbate, 0.1 M hydrogen peroxide, and 150 microliters of phytochemical materials extracted from the plant. APX concentration was measured based on the reduction of ascorbate absorption within 1 minute at 290 nm wavelength. The concentration of enzyme was calculated using the extinction coefficient (ϵ = 2.8 mmol^-1^cm^-1^) through the following formula [46].


A=ϵbc

A: read absorbance equivalent,

b: Cuvette length,

c: the concentration of the H_2_O_2_

The concentration of enzyme was calculated as phytochemical materials unit based on the amount of total protein (mg) present in 50 microliters of extract [obtained by the Bradford method (Bradford, 1976)] in one minute. One phytochemical materials unit of APX is the amount of phytochemical materials that oxidize one millimol of ascorbic acid in one minute (Nakano and Asada, 1981).

### Catalase activity

CAT activity was measured by a spectrophotometric method based on reducing hydrogen peroxide absorption in 30 seconds at 240 nm wavelength. The reaction mixture contained 50 mM potassium phosphate buffer with an acidity of 7, 15 mM H_2_O_2,_ and 100 microliters of phytochemical materials extract. The reaction was started by adding H_2_O_2,_ and the decrease in absorbance was measured in 30 seconds. The concentration of enzyme was calculated using the extinction coefficient (ϵ = 0.28 mmol^-1^cm^-1^) using the below formula [47];


A=ϵbc

A: read absorbance equivalent,

b: Cuvette length,

c: the concentration of the H_2_O_2_)

The concentration of enzyme was calculated as phytochemical materials unit based on the amount of total protein (mg) present in 100 microliters of extract (obtained by Bradford method (Bradford, 1976)) in one minute. One phytochemical materials unit of CAT is the amount of phytochemical materials that break down one mmol of H_2_O_2_ in one minute.

### Measurement of MDA

To measure malondialdehyde in chickpea leaves, 0.25g of leaf was ground with 5ml of 0.1% TCA and centrifuged. 250µL of supernatant was mixed with 1ml of a solution containing 20% TCA and 0.5% TBA. The mixture was heated at 95°C for 30 minutes, cooled in ice, and re-centrifuged. The absorbance was measured at 532nm, and non-specific dye absorbance at 600nm was subtracted. A specific equation was then used to calculate the malondialdehyde content. This method provided a precise quantification of malondialdehyde, an key indicator of oxidative stress, in chickpea leaf samples (Heath and Packer, 1968).


$$\text{MDA}({\text{umolg}}^{-1}\text{FW})=\frac{(\text{A}532-\text{A}600)\times \text{W}}{116}\times 1000$$

A532 = spectrophotometer-read absorption at 532 nm wavelength,

A600 = spectrophotometer-read absorption at 600 nm wavelength,

W= the weight of the Leaf sample used.

### Measurement of proline

To measure proline concentration, a 0.5g leaf sample was mixed with 10ml of 3% sulfosalicylic acid, homogenized, and filtered using a Watman N2 filter. 2ml of this filtrate was combined with 2ml ninhydrin reagent and 2ml acetic acid, heated at 100°C for an hour, and then quickly cooled in an ice bath. Next, 6ml toluene was added and stirred vigorously. After allowing it to settle for two hours, the upper phase was analyzed using a spectrophotometer at 520nm. A specific formula was then used to calculate the proline concentration, providing a precise method for quantifying proline in leaf samples (Bates et al., 1973).


$$\text{Proline}({\text{µM g}}^{-1}\text{fresh wt}.)=\frac{\text{M}\times \text{T}\times \text{W}}{115.5}\times 1000$$

Where;

M = the number read with the spectrophotometer;

T= the volume of toluene used (it was 4 ml);

W= leaf fresh weight.

### Data analysis and analysis

Statistical analysis was performed using Duncan’s Multiple Range Test at 1% and 5% significance levels, along with the mean square method for variance estimation. To ensure reliability and validity, the study incorporated randomization, multiple replications, control groups, and checks for data normality and variance homogeneity. Statistix vertion 10 software was used to analyze data and ANOVA, while Excel software was utilized for charts creation. Average data comparison were performed using the Duncan test at 1% and 5% probability levels.

## Results

**Figure 2:**
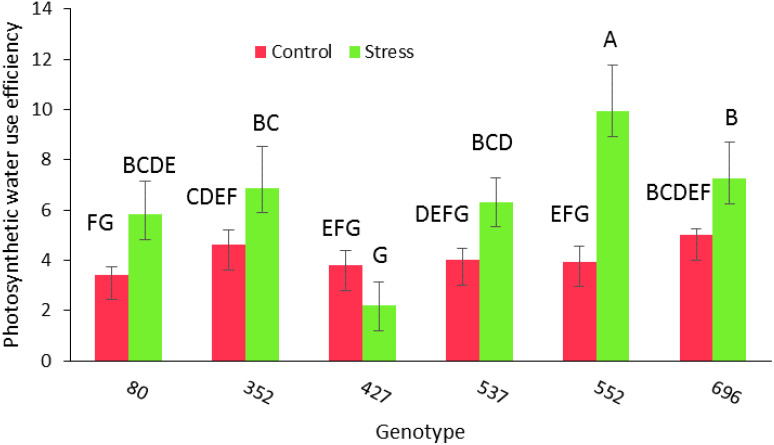
PWUE among different chickpea genotypes under drought stress and normal conditions. Genotypes with the same letters within the range indicate no significant difference (*p* ≤ 0.01).

Analysis of variance for morphological and phytochemical data showed no significant simple effect of drought stress and the interaction of the genotype at different levels of drought stress for the traits of the number of main branches and the number of lateral branches. However, there were significant differences in morphological characteristics including root diameter, CO_2_, EC, mesophyll conductivity (MC), photosynthetic rate(PR), PWUE, transport rate, number of branches, total root length, length of the upper part of the root (35 cm down from the soil surface), length of the lower part of the root (the second 35 cm from the middle of cylinder), length of the stem, root diameter, RWC, root dry weight, shoot dry weight, MSI, rootDW/ShootDW, root diameter (lower part), root diameter (upper part), chlorophyll content (SCMR) and leaf area (*p ≤* 0.01) (**Table 2**). Duncan**’**s mean comparison test showed that the highest amount of PWUE trait (9.94) occurred in the MCC552 genotype under drought stress conditions. The MCC696 genotype had the following position with 7.25 (**Figure 2**).

**Table 2:** Analysis of variance for morphological traits of chickpea genotypes.

Source	DF	MS			
		Root Diameter	Total root Length	Down root Length	Up root Length
Genotype (G)	5	0.007922^**^	2E+10^**^	1.476E+8^**^	2.163E+10^**^
Drought (D)	1	0.005539*	1.662E+10^**^	7.065E+8^**^	2.418E+10^**^
G * D	5	0.005732^**^	2.31E+10^**^	5.125E+7^*^	2.223E+10^**^
Error	24	1.01E-03	6.10E+07	1.69E+07	4.34E+07
Total	35				
Source	DF	MS			
		Root Diameter	Total root Length	Down root Length	Up root Length
Genotype (G)	5	51052.7^**^	0.20248^**^	1.60273^**^	1400.63^**^
Drought (D)	1	15376^**^	0.16947^**^	6.4009^**^	7290.04^**^
G * D	5	30505.3^**^	0.15897^**^	0.81564^**^	577.41^**^
Error	24	1238.1	0.00886	0.17008	19.37
Total	35				
Source	DF	MS			
		Root Diameter	Total root Length	Down root Length	Up root Length
Genotype (G)	5	1140.55^**^	0.00629^**^	11.886^**^	358.69^**^
Drought (D)	1	4272.85^**^	0.04427^**^	556.016^**^	1294.25^**^
G * D	5	365.56^**^	0.00781^**^	35.873^**^	37.08^*^
Error	24	69.49	0.00172	1.792	13.93
Total	35				
Source	DF	MS			
		Root Diameter	Total root Length	Down root Length	Up root Length
Genotype (G)	5	0.00489^**^	21.824^**^	3550^**^	
Drought (D)	1	0.02028^**^	467.633^**^	122325^**^	
G * D	5	0.00647^**^	15.528^*^	3050^**^	
Error	24	0.00099	4.016	97	
Total	35				

*, **, ***, respectively, Significant at the 0.05 (p < 0.05), 0.01 (p < 0.01), 0.001 (p < 0.001) level.

**Figure 3:**
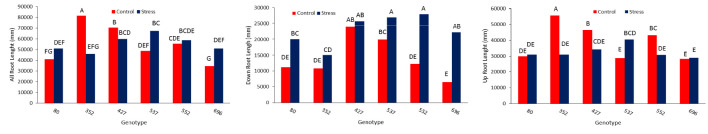
Comparison of different chickpea genotypes based on the total length of the roots **(A)**, the length of the lower root section **(B)**, and the length of the upper root section **(C)**. Genotypes with the same letters within the range indicate no significant difference (*p* ≤ 0.01).

**Figure 4:**
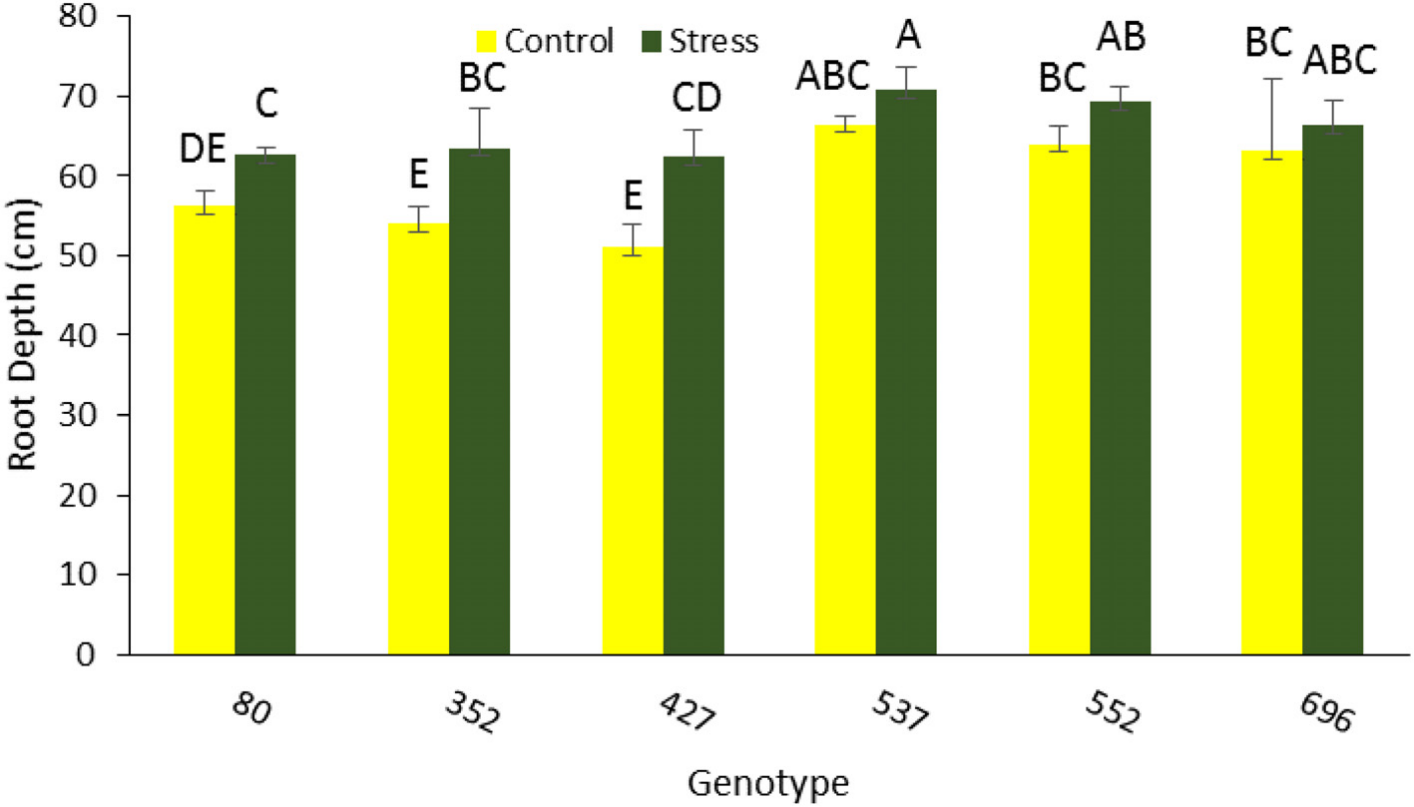
Root depth changes among different chickpea genotypes. Genotypes with the same letters within the range indicate no significant difference (*p* ≤ 0.01).

**Figure 5:**
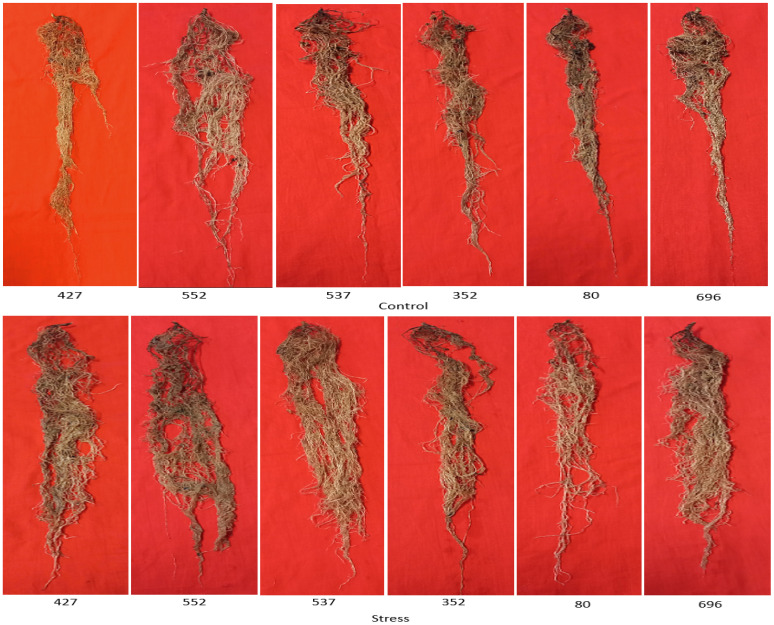
Represents whole roots washed among various chickpea genotypes under normal conditions (Up) and drought stress (down).

The highest average total root length was 67,300 mm for MCC537, 58,700 mm for MCC552, and 51,100 mm for MCC696 under drought stress conditions (**Figure 3A**). The highest total length of the roots in the lower part, with a total of 27944, 26941, and 22182 mm, was related to MCC552, MCC537, and MCC696 genotypes, respectively (**Figure 3B**). The highest length of roots in the upper part, 31,105 and 30,790 mm, was observed in the MCC537 and MCC552 genotypes, respectively (**Figure 3C**). The depth and penetration of the roots in the soil have increased in all chickpea genotypes under stress conditions compared to normal conditions. In addition, the soil’s highest root depth (70.83 cm) was related to the MCC537 genotype, followed by the MCC552 genotype with 69.36 cm (**Figures 4**, **5**).

In the present research, the root diameter increased only in two genotypes, including MCC352 and MCC552, under drought stress conditions compared to normal conditions. In contrast, the average root diameter in the MCC352 genotype has changed from 0.47 mm to 0.49 mm and in the MCC552 genotype from 0.43 to 0.44 mm due to stress condition (**Figure 6A**). Except for the MCC552 and MCC352 genotypes, the average root diameter in the lower part was reduced in the rest of the genotypes (**Figure 6B**). Notably, except for the MCC552 genotype, the average root diameter in the upper part was decreased in other genotypes. Also, in the MCC552 genotype, the average root diameter in the upper part was increased from 0.43 to 0.46 mm (**Figure 6C**).

**CBAFigure 6:**
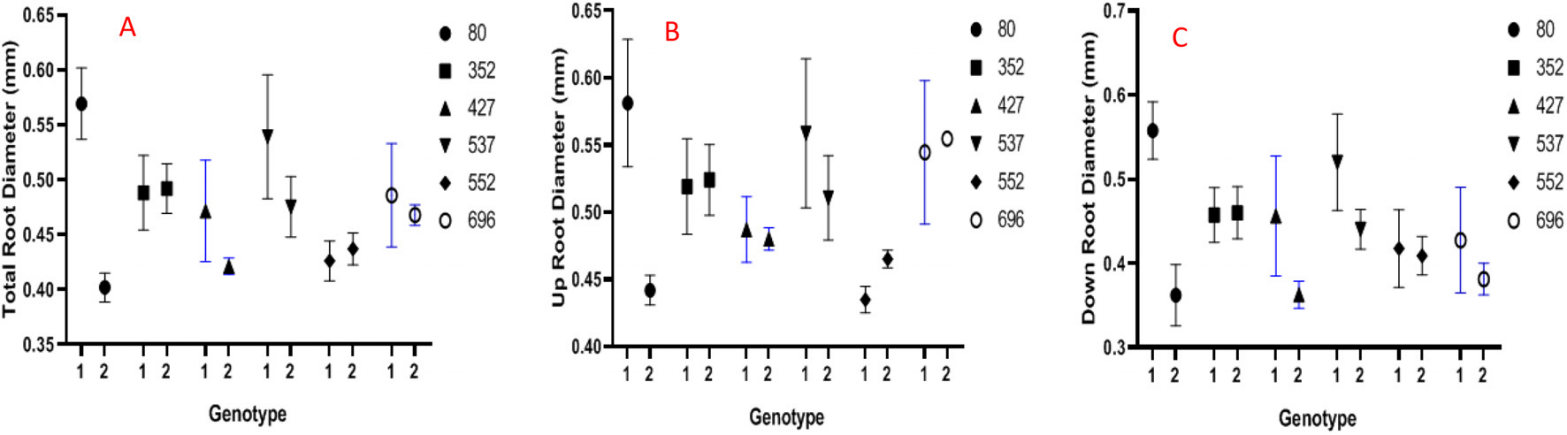
Evaluation of different chickpea genotypes based on the traits of the average diameter of the whole roots **(A)**, the average diameter of the upper part of the roots **(B)**, and the average diameter of the lower part of the roots **(C)** (*p* ≤ 0.01). 1= Control, 2= Stress.

**Figure 7:**
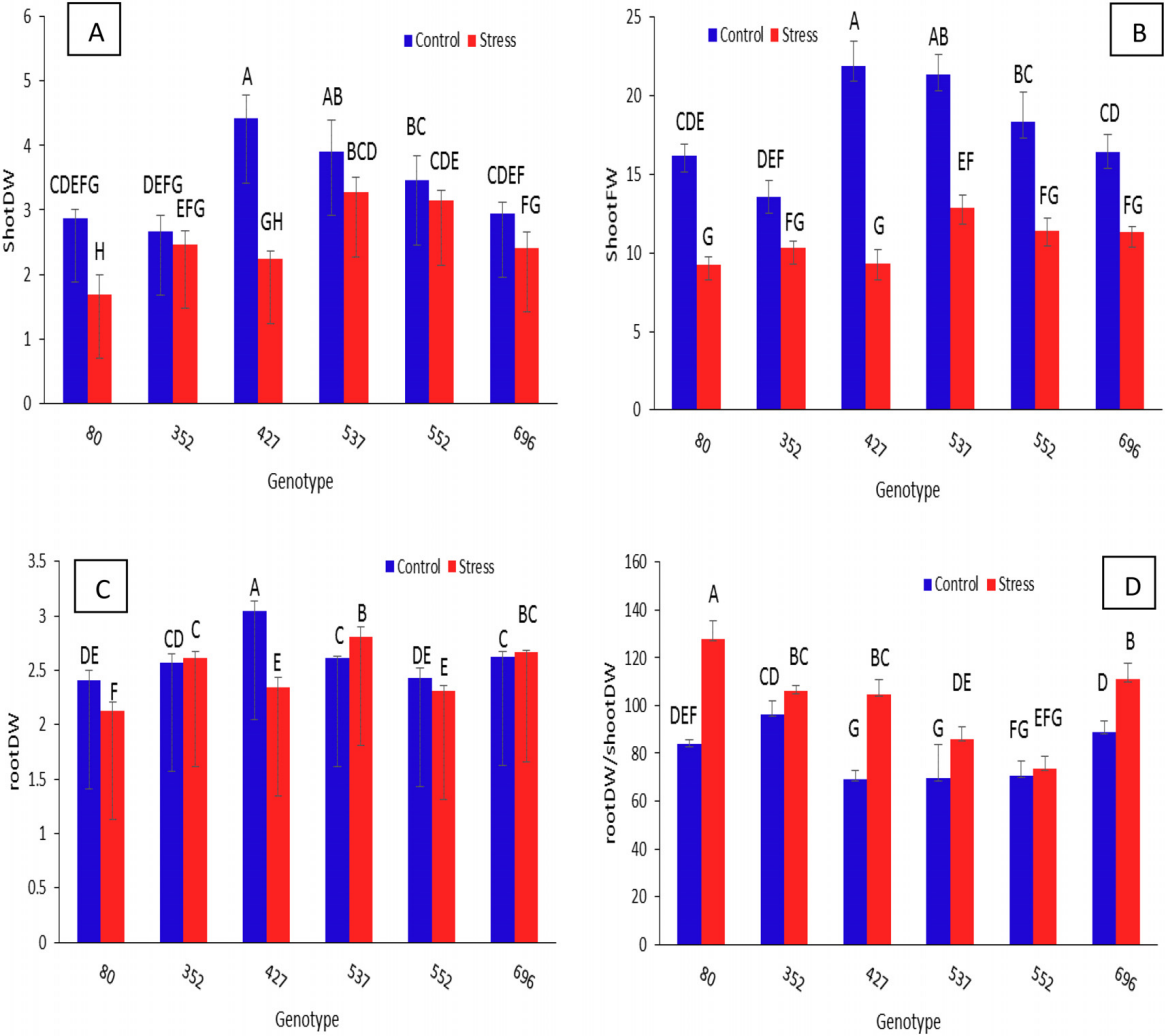
Assessment of different chickpea genotypes based on the shoot dry weight **(A)**, shoot fresh weight **(B)**, root dry weight **(C)**, and RootDW^*^/ShootDW ratio **(D)**. Genotypes with the same letters within the range indicate no significant difference (*p* ≤ 0.01). ^*^Dry weight (DW).

The shoot dry weight in all genotypes under stress has decreased significantly compared to normal conditions (**Figure 7A**). As a result, in the present research, the amount of leaf area for each genotype under stress conditions was divided by the same amount under normal conditions, and the genotype with the least reduction was considered. According to the data, the genotype MCC352 had the highest shoot dry weight ratio in stress conditions compared to normal conditions, followed by MCC552 and MCC696 with ratios of 0.92, 0.81, and 0.9, respectively. For the ratio of root dry weight in stress conditions compared to normal conditions, the highest values were observed in genotypes MCC696, MCC537, MCC352, and MCC552, with rates of 1.07, 1.01, 1.01, and 0.95, respectively (**Figure 7C**). The genotype MCC80 had the highest root/shoot dry weight ratio, followed by MCC696 with a ratio of 111/06 (**Figure 7D**). As for the fresh weight ratio in stress conditions compared to normal conditions, the highest values were observed in genotypes MCC352, MCC696, and MCC552, with ratios of 0.76, 0.69, and 0.62, respectively (**Figure 7B**).

**Figure 8:**
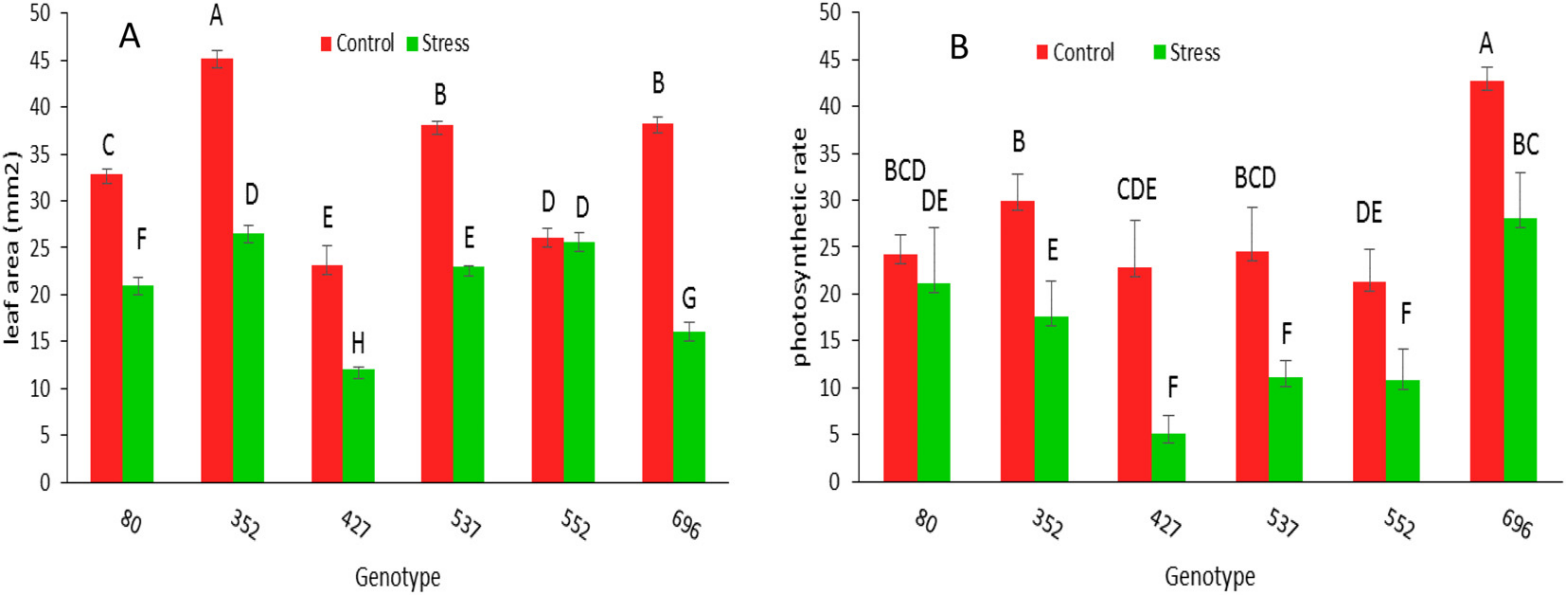
Leaf area (mm^2^) **(A)** and photosynthetic rate (µmol m-2s-1) **(B)** changes of different chickpea genotypes under normal and stress conditions. Genotypes with the same letters within the range indicate no significant difference (*p* ≤ 0.01).

**Figure 9:**
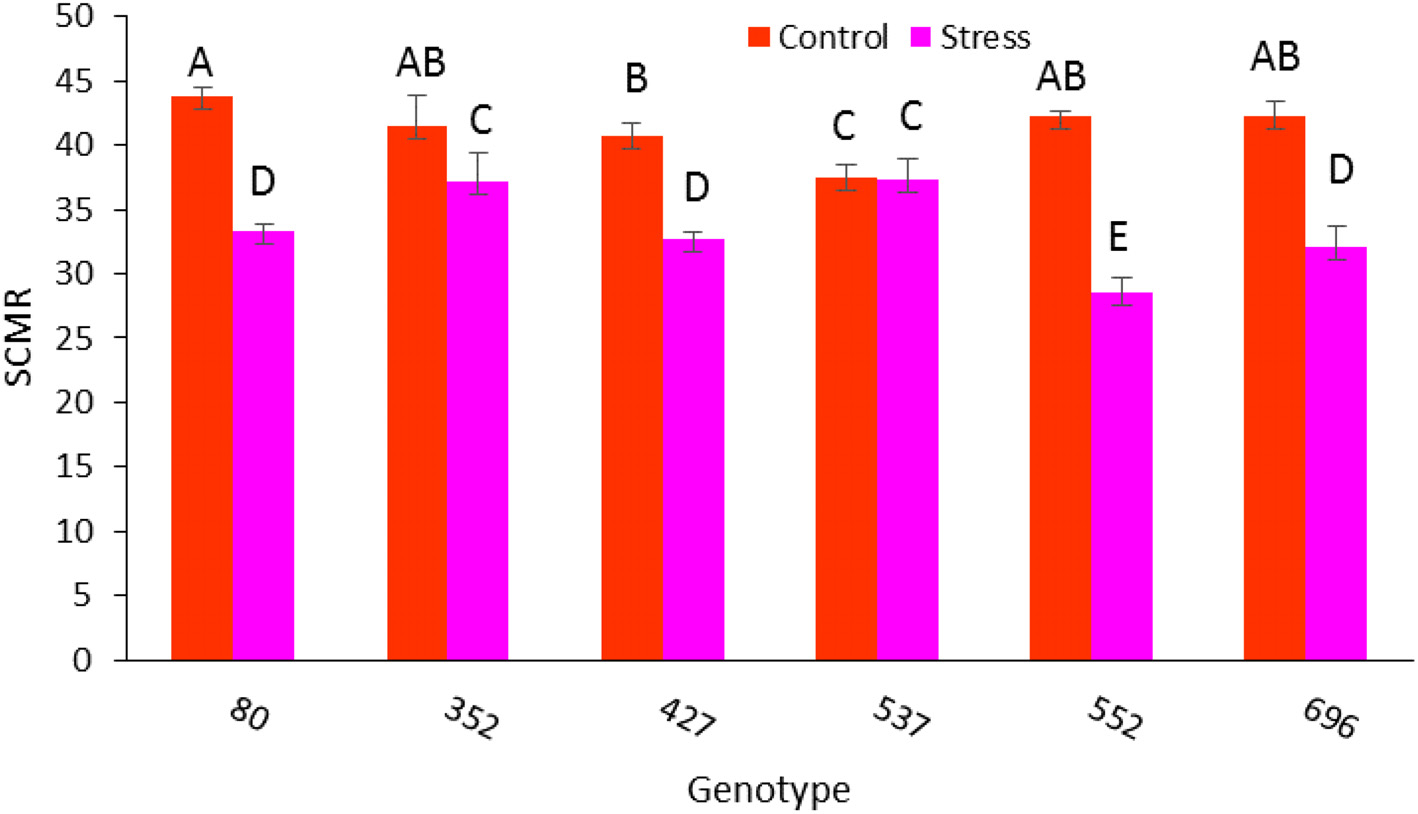
Chlorophyll content (SCMR((SPAD chlorophyll meter reading)) changes in different chickpea genotypes. Genotypes with the same letters within the range indicate no significant difference (*p* ≤ 0.01).

The leaf area under stress in all genotypes, except 552 genotypes, has decreased significantly compared to normal conditions (**Figure 8A**). The photosynthetic rate ratio under drought stress conditions compared to normal conditions in MCC80, followed by MCC696, MCC352, and MCC522 genotypes, was 0.86, 0.65, 0.58, and 0.50, respectively (**Figure 8B**). According to the ratio of SCMR under drought stress compared to normal conditions, the highest amount of SCMR occurred in MCC537 and then MCC352, respectively, with the amounts of 0.99 and 0.89 (**Figure 9**).

**Figure 10:**
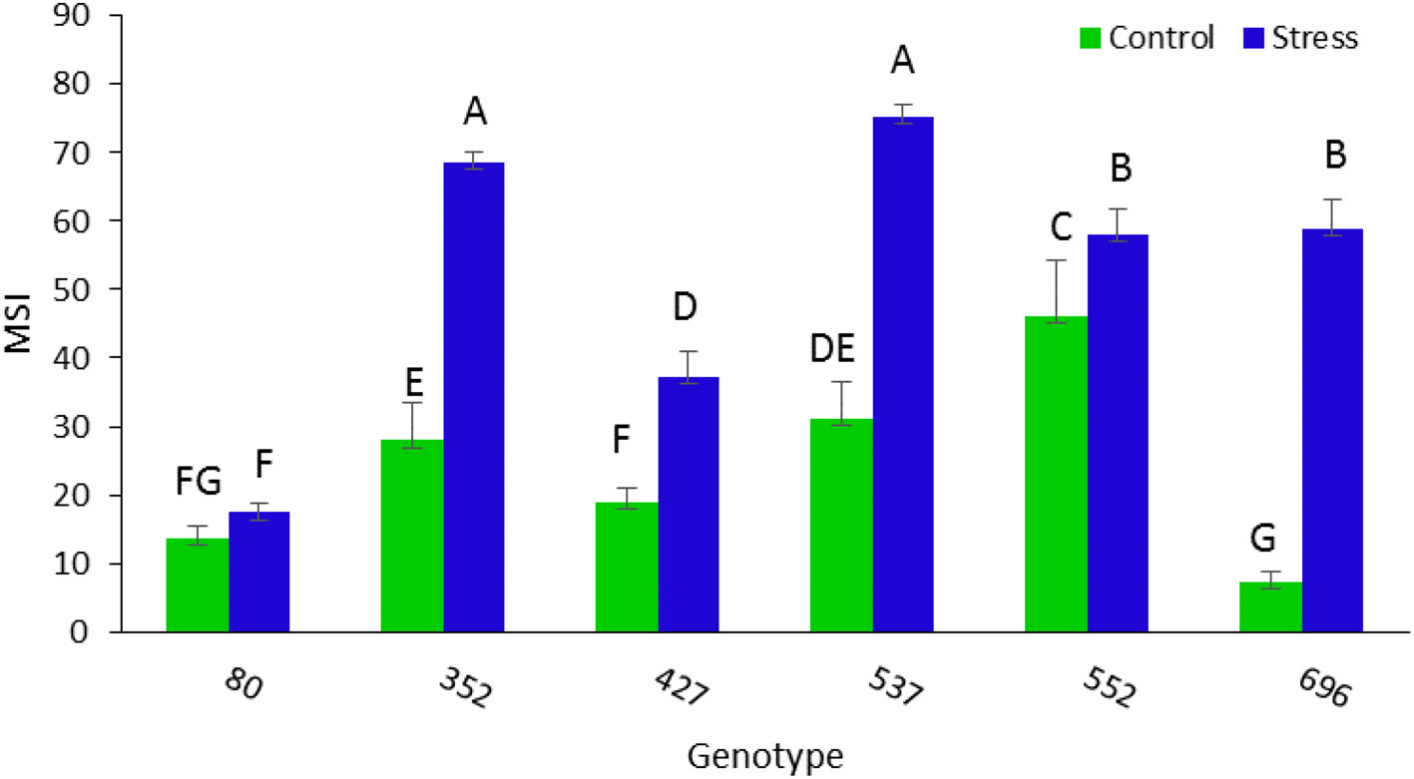
Evaluation of different chickpea genotypes based on MSI. Genotypes with the same letters within the range indicate no significant difference (*p* ≤ 0.01).

**Figure 11:**
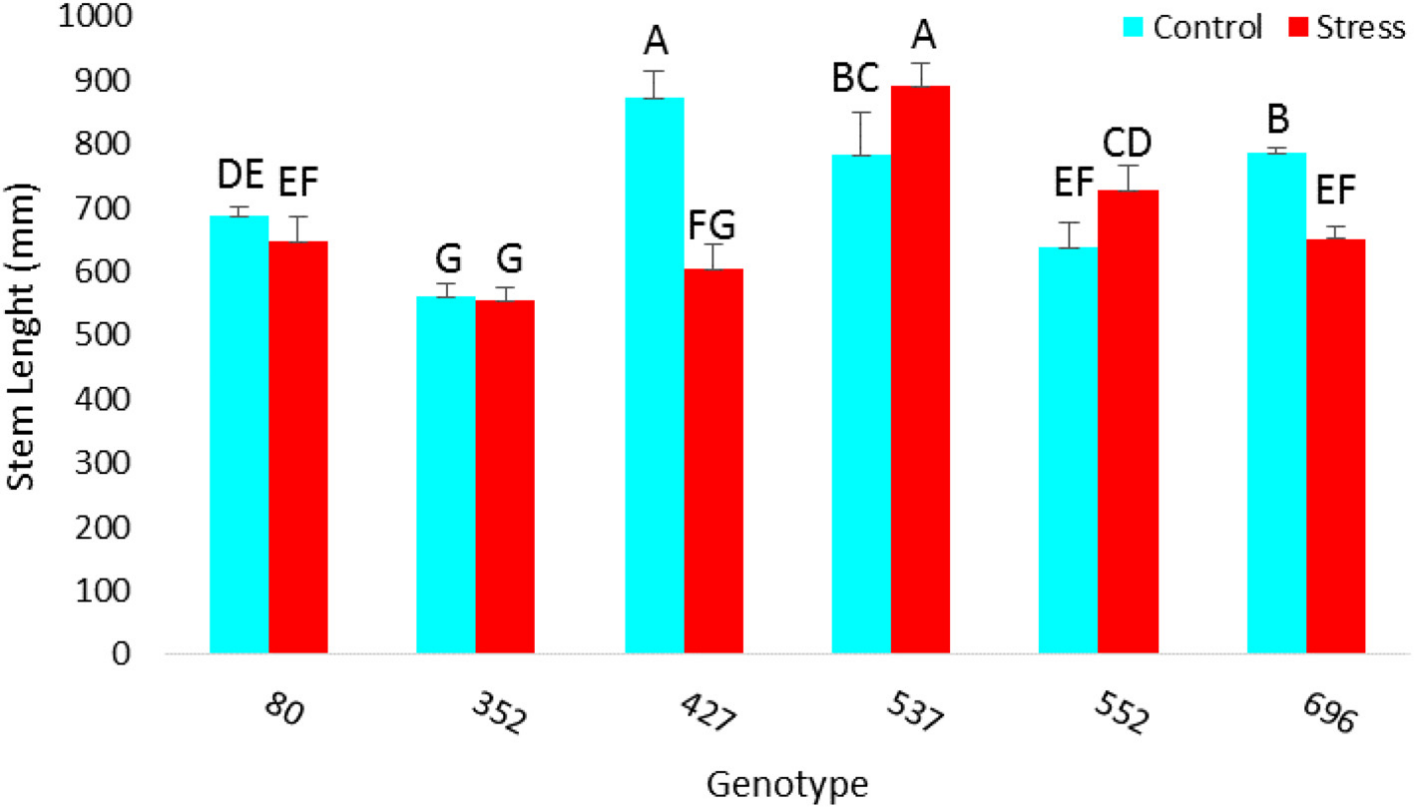
Evaluation of different chickpea genotypes based on each genotype’s average length of stems. Genotypes with the same letters within the range indicate no significant difference (*p* ≤ 0.01).

The highest amount of MSI was obtained in MCC537 (75.32), MCC352 (68.7), followed by MCC552 (58.8) and MCC696 (57.9) genotypes (**Figure 10**). The highest average length of stem was observed in the MCC537 and MCC552 genotypes, with 892.6 and 728 mm, respectively (**Figure 11**).

**Figure 12:**
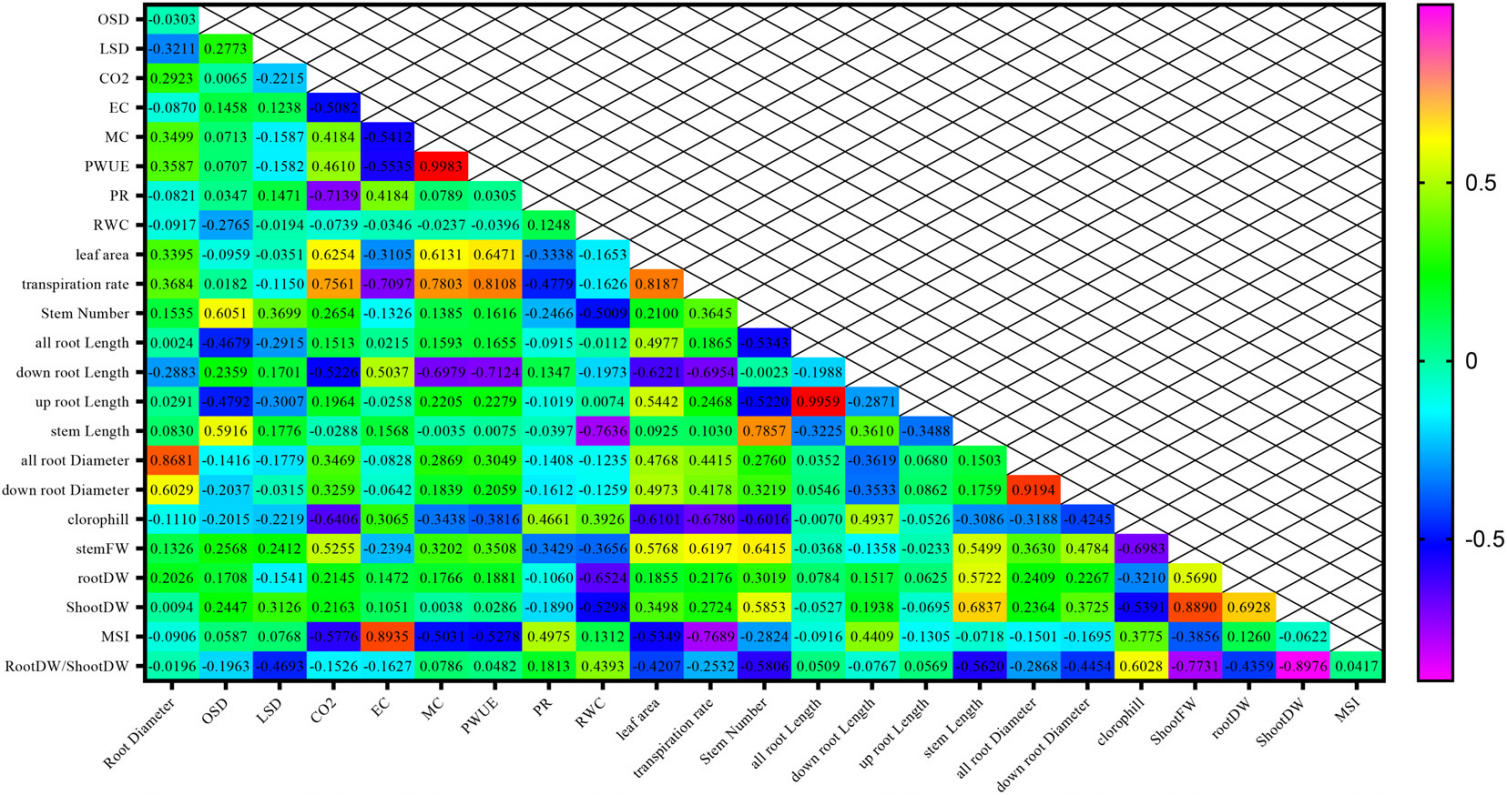
Correlation between morpho-physiological traits among different chickpea genotypes.

The highest correlations of 0.998, 0.995, and 0.919 were respectively detected between mesophyll conductance and photosynthetic rate traits, total root length traits with the length of the upper part of the root, and the traits of average root diameter with the average diameter of the lower part of the root (**Figure 12**).

**Figure 13:**
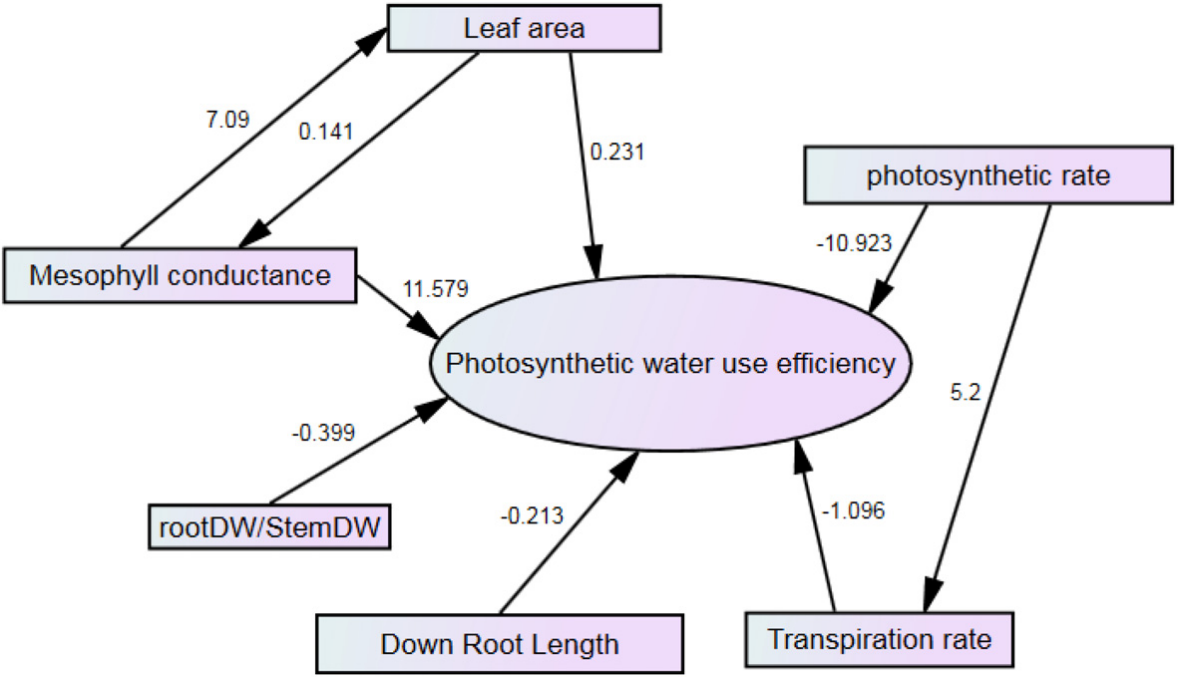
Path analysis of the effect of studied traits on PWUE.

Stepwise regression showed that seven traits could affect PWUE. Traits such as mesophyll conductance and leaf area directly and positively affect PWUE. Mesophyll conductance, with 11.579, has the highest positive rate. However, the photosynthetic rate has the most significant effect on PWUE and indirectly affects the transpiration rate. The photosynthetic rate was the most negative effect on PWUE (**Figure 13**).

PWUE = 1091.79 Mesophyll conductance – 2.523 photosynthetic rate + 0.008 leaf area – 1.117 transpiration rate -0.06451 lower root length – 0.048 root DW/shoot DW

**Figure 14:**
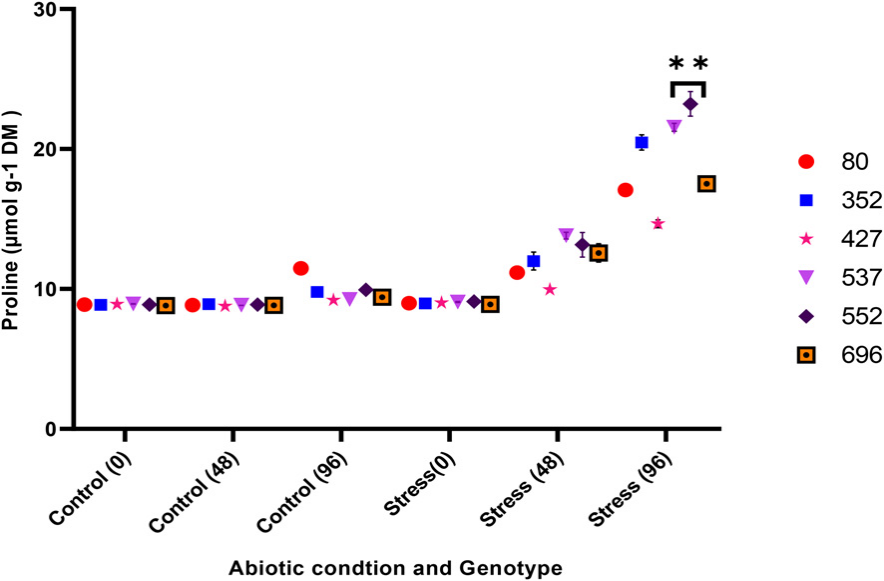
Changes in proline content of chickpea genotypes at various drought stress time points (0, 48, and 96 hrs) (*p* ≤ 0.01). **, Significant at the 0.01 (p < 0.01) level.

**Figure 15:**
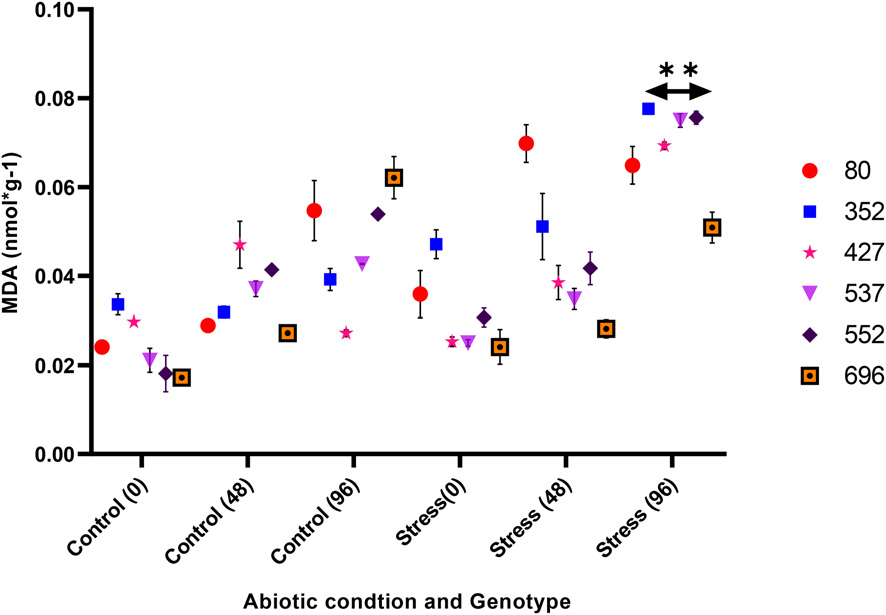
Different chickpea genotypes evaluation based on the MDA at various drought stress time points (0, 48, and 96 hrs) (*p* ≤ 0.01). **, Significant at the 0.01 (p < 0.01) level.

**Figure 16:**
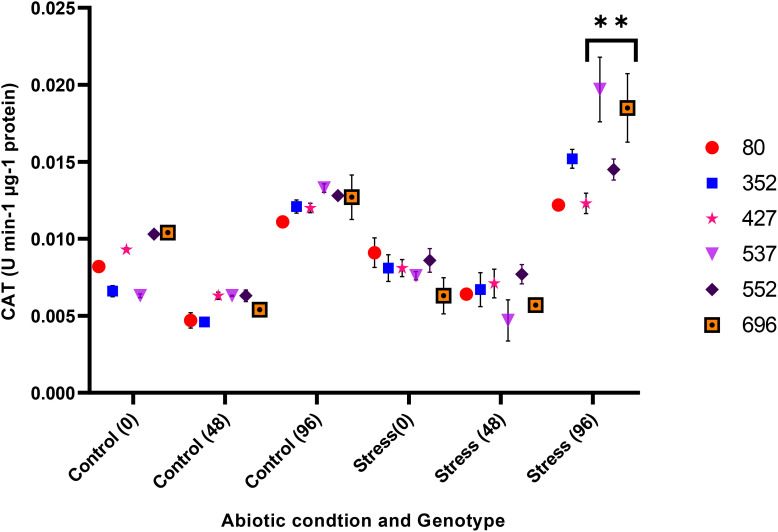
Different chickpea genotypes evaluation based on the CAT at various drought stress time points (0, 48, and 96 hrs) (*p* ≤ 0.01). **, Significant at the 0.01 (p < 0.01) level.

The analysis of variance for the phytochemical data showed that the simple effect and interactions of the genotype, drought stress, and time were significant on the proline, POD, MDA, CAT, and GPX traits (*p* ≤ 0.01) (**Table 3**). The highest proline content was observed in the MCC552 genotype. Meanwhile, with the increase in the time of stress, the amount of the proline was also increased (**Figure 14**). The lowest levels of MDA occurred in the genotype MCC696. Meanwhile, with the increase in the time of stress, the rate of the MDA has also increased (**Figure 15**). The highest rate of CAT was observed in the genotypes MCC696, followed by MCC537 and MCC552. A similar pattern was also happened in CAT phytochemical materials with the increase of stress time (**Figure 16**).

**Table 3:** Analysis of variance for the phytochemical traits of chickpea genotypes.

Source	DF	SS	MS				
			GPX	CAT	MDA	POD	prolin
Genotype (G)	5	5.50E-05	1.10E-05^**^	1.99E-05^**^	3.88E-04^**^	2.21E-05^*^	9.413^*^
Drought (D)	1	3.61E-06	3.61E-06^**^	3.70E-06*	4.35E-03^**^	1.25E-05^**^	487.936^**^
Time (T)	2	2.81E-04	1.40E-04^**^	7.26E-04^**^	8.24E-03^*^	4.55E-04^**^	285.729^*^
G*D	5	1.08E-05	2.16E-06^**^	1.39E-05^**^	3.53E-04^*^	6.22E-06^*^	11.541^**^
G*T	10	1.04E-04	1.04E-05^**^	1.68E-05^**^	2.04E-04^**^	1.40E-05^**^	3.904^**^
D*T	2	1.03E-05	5.13E-06^**^	1.48E-05^**^	6.19E-04	5.45E-06^**^	198.28^**^
G*D*T	10	2.36E-05	2.36E-06^**^	7.87E-06^**^	4.17E-04^**^	4.70E-06^**^	5.058^**^
Error	72	2.89E-06	4.01E-08^**^	6.21E-07^**^	9.79E-06^*^	8.17E-08^**^	0.088^**^
Total	107	4.90E-04					

*, and **, respectively, Significant at the 0.05 (p < 0.05), 0.01 (p < 0.01) level.

**Figure 17:**
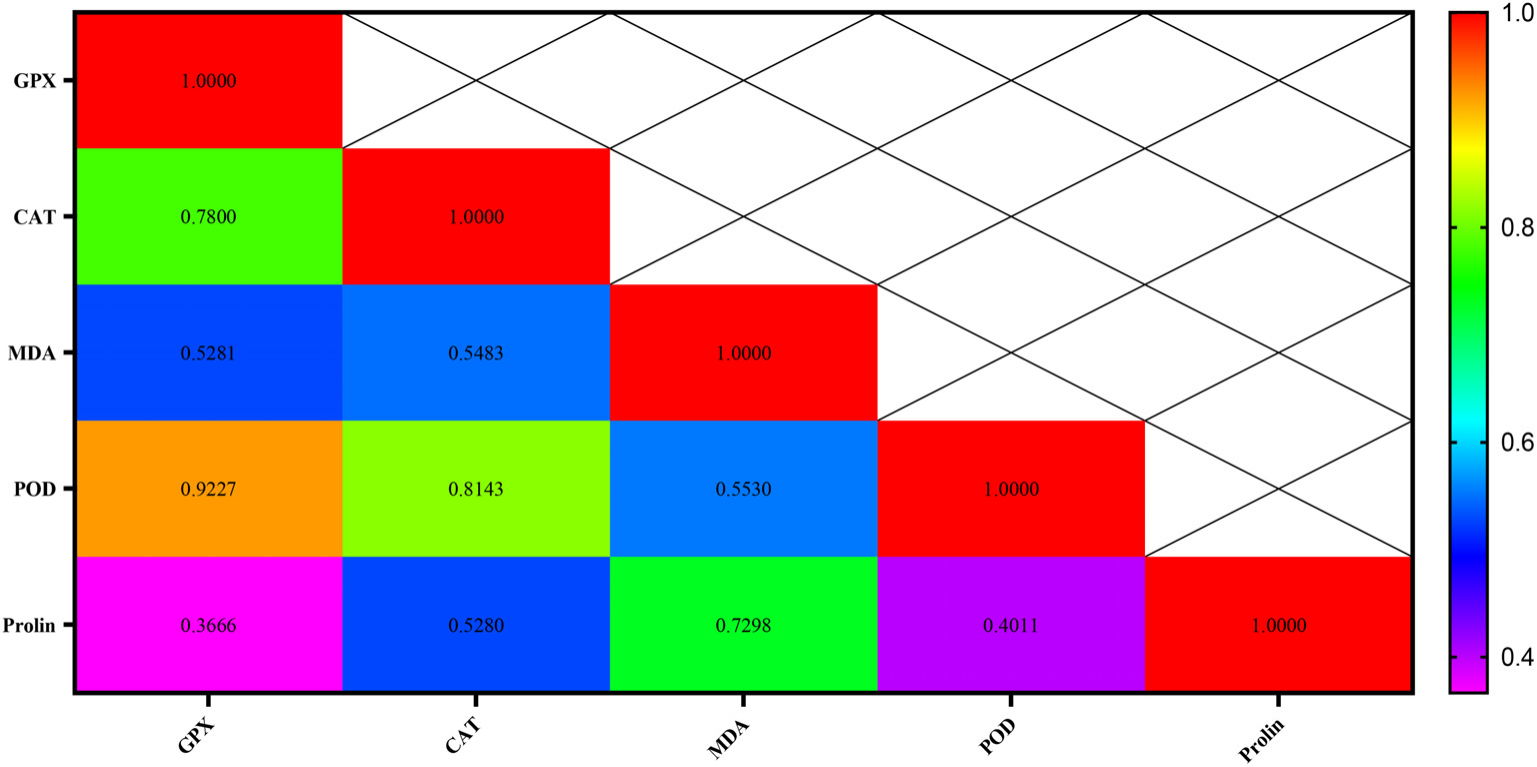
Correlation between antioxidant enzymes (GPX, CAT, and POD), MDA, and proline in chickpea genotypes.

The highest correlation rates were observed with 0.92 and 0.81 between the POD and GPX traits, followed by POD and CAT, respectively. The lowest correlation rate (0.36) was also between proline and GPX attributes (**Figure 17**).

## Discussion

In the current research, it was found that drought stress increased proline and CAT. MDA was also affected by drought and increased with the increasing time of applying drought stress. In research (Raheleh et al., 2012), tolerant genotypes (MCC392 and MCC877) and sensitive (MCC68 and MCC448) have been compared in terms of proline, MDA, CAT, APX, peroxidase, and superoxide dismutase at seedling, flowering, and poding stages under drought stress (25% WHC),. Their results showed that drought stress caused a significant increase in proline at the flowering and podding stages, as well as an increased CAT activity in all three stages. By contrast, the effects of drought stress on APX, peroxidase, and MDA have not been significant. At the flowering stage, it has been indicated that the tolerant genotype had higher CAT activity and proline than the sensitive genotypes. It is noted that the activity of CAT, superoxide dismutase, and proline can be effective indicators in identifying drought-tolerated chickpea genotypes (Raheleh et al., 2012).

It has been shown that the MCC696 genotype has had the highest amount of proline compared to the MCC588, MCC877, and MCC776 genotypes (Abrishamchi et al., 2012). However, in the current research, MCC552 and then MCC537 genotypes were more competitive than MCC696 in proline content under drought stress conditions. The other study also reported that proline content was higher in tolerant chickpea cultivars compared to sensitive ones due to drought stress. The current research also found that drought stress increased the antioxidant enzymes (GPX, CAT, and POD), MDA, and proline. One of the reliable indicators for selecting plants under drought conditions can be the accumulation of proline in different plant organs; the accumulation of proline could be directly related to the plant’s drought tolerance (Mamnoei and Seyed Sharifi, 2010). Proline corrects the negative effect of sodium chloride salt and water stress on carbon fixation and can moderate the reduction of Rubisco enzyme activity under such conditions (Mamnoei and Seyed Sharifi, 2010).

Proline can enhance the efficiency of photosynthetic water use in plants. Studies have shown that applying exogenous proline can positively affect plant growth, leaf chlorophyll content, leaf relative water content, and overall photosynthetic performance, especially in stressful conditions such as salt stress (Hayat et al., 2012; El Moukhtari et al., 2020; Spormann et al., 2023). Research has shown that applying exogenous proline can boost photosynthetic attributes in plants, including net photosynthesis, stomatal conductance, transpiration rate, and chlorophyll content. This improves plant growth and performance, especially in challenging environments (El Moukhtari et al., 2020; Spormann et al., 2023). In addition, proline is involved in maintaining redox balance, improving photosynthetic efficiency, and ensuring a healthy nutrient balance in plants under stress (Spormann et al., 2023). Increasing proline concentration in plants subjected to water deficit can help reduce water loss from plant cells, improving water use efficiency (Medina et al., 2023).

The effect of proline on drought-tolerant candidate chickpea genotypes is significant in enhancing stress tolerance. Research indicates that proline accumulation in chickpeas is particularly active against drought stress, aiding stress tolerance mechanisms (Vessal et al., 2020). Proline accumulation in chickpea genotypes has been shown to contribute significantly to their ability to withstand drought and heat stress conditions (Kaushal et al, 2011).

In drought stress conditions, the total root length, depth of root penetration, and expansion in the lower parts of the soil are significant traits for evaluating plants suffering from drought stress. Root traits, such as root length density (RLD) in relatively shallow soil layers and root depth (RDp) in chickpeas, can positively influence seed yield in drought-prone environments by delaying dehydration (Kashiwagi et al., 2005; Gaur et al., 2008). In the present study, MCC552 and MCC696 had the highest root biomass of all the genotypes studied by chickpeas. The ratio of root dry weight to shoot dry weight in chickpea genotypes has been significantly influenced by genotype and drought stress. The MCC80 genotype had a root-to-shoot dry weight ratio of 1.36, while the MCC552 genotype had a ratio of 0.72 (Ganjeali et al., 2011c). In the present study, the lowest ratio of root-to-shoot dry weight belonged to the MCC552 genotype. However, this decrease in the ratio can be due to the weight loss of the shoot organs, as the MCC552 genotype has managed to maintain its yield in drought-stress conditions (Ganjeali et al., 2011c). It has been reported that the increase in the root/shoot organ ratio is mainly related to the more significant reduction in the shoot dry weight compared to the root dry weight in drought stress conditions (Saxena, 2003).

A comparative physiological and proteomic analysis examined the stress response of two chickpea species, *C. reticulatum* and *C. arietinum*, under drought conditions (Çevik et al., 2019). The results demonstrate that drought stress decreased root length and leaf water content while increasing the free proline content in both species. It has been shown that the effect of drought stress in *C. arietinum* was more than in *C. reticulatum*, mainly due to the photosynthetic capacity (Çevik et al., 2019). The present study also found that drought stress has increased the leaf water content as well as the phytochemicals such as GPX, CAT, POD, MDA (physiological metabolite), and proline.

Plants have a complex phytochemical defense system to reduce oxidative damage caused by ROS, including non-enzymatic and enzymatic phytochemicals. CAT plays an important role in the phytochemical system, converting hydrogen peroxide (H_2_O_2_) into oxygen and water. This enzyme is sensitive to non-biological stress conditions and is a stress marker. CAT activity increases under water limitation (Dos Santos et al., 2013). The CAT enzyme has been shown to negatively correlate with photosynthesis, stomatal conductance, and transpiration (Dos Santos et al., 2013).

High levels of electrolyte leakage and MDA accumulation are commonly recognized as signs of injury caused by stress. Stresses, always accompanied by an increase in electrolyte leakage from the cell, lead to a decrease in membrane integrity. In this study, the researchers found that interrupting irrigation significantly reduced membrane stability. This was evident through the increased electrolyte leakage and MDA content in the leaf. Any factor that can regulate the activity of phytochemicals to modulate MDA accumulation to maintain cell membrane stability can play an influential role in reducing the effect of drought stress on morphophysiological and phytochemical indicators (Ahmed et al., 2019). Similar results have also been observed in wheat, corn, and rice (Raza et al., 2007; Kathuria et al., 2009; Anjum et al., 2017). Increased CAT activity and proline accumulation during periods of low water availability in the soil indicate that the MCC552 genotype would have an efficient protective mechanism to survive drought stress conditions (Dos Santos et al., 2013).

It is expected that mesophyll conductance and leaf area would increase the PWUE. In the current research, it was also found that MDA, although very little, had a positive effect on PWUE. The MDA compound can impact photosynthetic water use efficiency in plants. Research has shown that MDA, which reflects lipid peroxidation in plant cells and responses to external stress, can influence plant biomass, photosynthesis, and lipids (Song et al., 2016). Additionally, studies have demonstrated that overexpression MDATG8i, a gene related to autophagy, can improve water use efficiency in plants by enhancing photosynthetic capacity and growth performance through optimized stomatal apertures and protection of the photosynthetic apparatus (Jia et al., 2021). Furthermore, elevated CO2 levels have been linked to changes in photosynthesis and phytochemical activity under drought stress, indicating a complex interplay between environmental factors and plant responses (Li et al., 2020). Studies have also explored the relationship between water use efficiency and photosynthesis in different plant species, highlighting the importance of factors like stomatal conductance, net photosynthetic rate, and water availability in influencing overall plant productivity (Niu et al., 2022; Wu et al., 2022).

The relationship between root dry weight and photosynthetic water use efficiency (PWUE) is complex. Research shows that water deficit can significantly impact root growth and shoot development, affecting several physiological processes, including growth, stomatal conductance, and photosynthesis. Additionally, this relationship is influenced by factors such as nutrient availability, plant species, and environmental conditions (Dinh et al., 2017; Khalil et al., 2020). Root pruning, which involves reducing root biomass, has improved water-use efficiency in maize by enhancing root water absorption (Yan et al., 2022). Studies have indicated that decreasing the root/shoot ratio through root pruning can significantly enhance grain yield and WUE (Yan et al., 2022). Root pruning can improve photosynthetic traits and root hydraulic conductivity, affecting water uptake and plant efficiency (Yan et al., 2022). However, the effects of root pruning on yield may vary depending on water conditions, with reports of increased grain yield under drought but not under sufficient water supply (Yan et al., 2022). Based on this, root growth under stress lowers photosynthetic water use efficiency.

The shoot, the leaves, and the height of the plant will decrease under the drought stress conditions. As a result, the plants are forced to use optimal water, thus increasing water consumption efficiency. In a study (Nezami et al., 2005), the effects of drought stress on some morphological traits (plant height, length and number of lateral branches, number of leaves, number of flowers and pods) among several chickpea genotypes have demonstrated that there has been a great diversity among genotypes. Some genotypes, such as MCC101 and MCC174, have responded better to stress conditions, and their growth indicators have been less affected by stress (Nezami et al., 2005). In the present study, the branch number and the number of leaves were affected by drought stress and were reduced in most chickpea genotypes. The chickpea height was also affected by drought stress, and it was found that the highest average length of the stems was obtained in the MCC537, followed by MCC552 genotypes.

Screening of 150 kabuli chickpea genotypes against drought stress showed a significant variation among genotypes in quantitative traits, and there have also been positive and very significant correlations between grain yield and stress-tolerance indexes (Ganjali et al., 2009). Due to the very high correlations of these indexes with yield in none-stress conditions, several genotypes were proposed as candidate genotypes for drought tolerance. In the present study, the MCC552 and MCC696 genotypes have been better in most measured traits under drought stress conditions and have a higher drought tolerance than others.

The present study also found that stress caused a reduction in mesophyll conductivity and photosynthetic rate. With the increase of drought stress, mesophilic conductivity was also affected more than stomatal conductivity, resulting in a reduction in the entry of CO2 into the stomata and consequently in limited carboxylation efficiency and CO2 consumption (Andalibi and Nouri, 2014).

By studying the effect of drought stress on the chlorophyll in the barley, it has been observed that with higher limitation of irrigation water, chlorophyll levels and phytochemical efficiency of photosystem II have shown a significant decrease due to optical inhibition (Mamnoei and Seyed Sharifi, 2010). The present study also found that the efficiency of photosynthesis in plants under drought stress was lower than in plants with normal growth conditions.

Chlorophyll SPAD values are usually lower when plants are under drought stress. This stress can cause a decrease in chlorophyll content due to various physiological and biochemical changes that happen in response to lack of water. When plants experience drought, their photosynthetic rates often decrease, which results in a decline in chlorophyll content. This decrease in chlorophyll content is usually accompanied by alterations in other physiological parameters, including relative water content (RWC), which drought stress can also impact (Qi et al., 2021; Arief et al., 2023; Pallavolu et al., 2023). In this research, the chlorophyll SPAD was reduced in stress conditions less than in normal conditions, similar to previous research.

*Ctenanthe setosa* plant under severe drought stress showed a reduction in leaf RWC from 94% to 74%. The decrease in leaf water potential and RWC has been associated with lower stomatal conduction and photosynthesis and, consequently, reduced yield (Saglam et al., 2008). The present study also found that RWC was less common in plants under stress than in plants with normal growth conditions. The decrease in leaf RWC can be due to decreased water absorption by the roots or greater evaporation from the stomata. High RWC in water limitation conditions can be associated with plant root system function and profound root growth for maintaining the inner water content of the plant (Andalibi and Nouri, 2014).

A plant’s relative water content (RWC) does not increase in drought conditions. It usually decreases when the plant is under drought stress. This is because drought results in a water deficit in the plant, causing a reduction in the water content of the leaves compared to their maximum water-holding capacity (Soltys-Kalina et al., 2016; Shariatmadari et al., 2017). Under normal conditions, the relative water content (RWC) in fully turgid leaves is typically around 98%. However, drought stress can lead to a significant decrease in RWC. For instance, a study demonstrated that drought stress resulted in a 170% reduction in RWC in chickpea plants under control conditions and a 97% reduction under priming conditions (Shariatmadari et al., 2017). Similarly, another study found that drought stress caused a decrease in potato plants’ RWC (Relative Water Content). Initially, the RWC values dropped to around 60% to 70% during wilting, and for severely dried leaves, the values plummeted to as low as 30% to 40% (Soltys-Kalina et al., 2016).

Photosynthetic rates under stress usually do not exceed high values (>20). Both heat stress and water stress inhibit photosynthesis. In the case of heat stress, photosynthesis experiences a significant decrease after 3 hours, and it can only partially recover after 12-24 hours of exposure to high temperatures (42°C). Prolonged heat stress reduces electron transport and photosystem damage and decreases photosynthetic capacity (Song et al., 2014). During periods of water stress, there is a consistent decrease in stomatal conductance (gs) and photosynthetic rate as the relative water content (RWC) decreases. At low RWC levels, the photosynthetic potential measured under saturating CO_2_, known as A pot, becomes increasingly inhibited. This inhibition suggests that metabolic factors play a significant role in limiting photosynthesis. Traditionally, it has been observed that as RWC decreases, photosynthetic rate and A pot approach or reach zero at approximately 40% RWC. However, elevated levels of CO_2_ can sometimes maintain the photosynthetic rate at A pot even as RWC decreases. It is important to note that in some instances, elevated CO2 becomes less effective in stimulating A as RWC decreases, indicating further inhibition of A pot (Lawlor, 2002). Although photosynthetic rates are generally high in unstressed conditions, they are significantly reduced when plants are exposed to heat and water stress. As measured by parameter A pot, plants’ photosynthetic activity becomes inhibited when the relative water content (RWC) is low or after prolonged exposure to high temperatures. It is important to note that photosynthetic rates do not exceed 20 under these challenging conditions (Lawlor, 2002; Song et al., 2014). In this research, the photosynthetic rates for all genotypes, except 696 genotypes, have been reduced, and the grade was less than 20, but the 696 genotypes had more than 20. This section of the experiment was repeated two times, and the results were the same; they were not changed. The authors could not find any reasons for this result.

A study of root morphological traits as appropriate criteria for selecting chickpea genotypes under drought stress indicated that in the flowering and pod formation, the interaction of stress and genotype was not significant on the length of the primary root. However, in the filling stage of the seeds, the drought stress has significantly reduced the length of the main root in most genotypes relative to the control condition. In addition, the ratio of root to shoot organs in response to drought stress has increased up to the flowering stage, which is related to the further decrease in the growth of shoot organs compared to roots at this stage. Still, this ratio has decreased after flowering (Ganjeali and Bagheri, 2010). In the current study, because of the measurement of root length, root dry weight, and shoot dry weight before flowering, the results demonstrated a similar positive ratio of root-to-shoot dry weight.

During drought stress, plants would grow their roots profoundly.

Absorbing water from different deep soil layers would reduce the roots’ diameter. In addition, plants forced to increase the growth length of their roots during drought stress, increase the average root diameter, or try to prevent the reduction of root diameter show more ability to deal with drought stress (Shirazi et al., 2016; Shahi et al., 2019; Fazeli-Nasab et al., 2022; Hoseini et al., 2022; Karimian et al., 2023). Studies show a positive correlation between root diameter, depth, and plant vigor under drought stress. A deep root system with a larger diameter is beneficial for plants like rice, wheat, and beans to acquire moisture from soil profiles efficiently. Root length densities and surface area are vital for water uptake in plants under drought conditions. Higher root surface area helps overcome hydraulic resistance in dry soil (Comas et al., 2013; Ye et al., 2018; Kim et al., 2020). In the present study, the length and diameter of the root were associated with the increase in drought stress duration. It has been reported that root growth is affected by the intensity of the plant’s stress, genotype, and phenological stage. In this regard, drought stress first increases root growth and then reduces it, but the decline in growth depends on the time of restriction of photosynthetic material from the roots (Saxena, 2003).

Drought resistance in a plant depends on various factors, including the plant species and its genotype, the age and size of the plant, the duration of the drought, and the environmental conditions. Plants have evolved different strategies to cope with drought stress, such as stomatal regulation, osmotic adjustment, and drought escape. Genetic factors, environmental conditions, and management practices can influence these strategies. Understanding these factors can help develop drought-resistant plants and improve agricultural productivity in drought-prone areas (Ullah et al., 2019; Abhilasha and Roy Choudhury, 2021; Vonapartis et al., 2022; Debnath et al., 2023). As a result, the selection of drought stress-resistant varieties should also be consistent with the selection of genotypes with high genetic potential in none-stress conditions (Ganjeali et al., 2011a).

Roots with specific traits like diameter, depth, length, and surface area can be critical in enhancing plant resilience to drought stress by improving water uptake efficiency and overall plant vigor. Understanding these root characteristics is essential for breeding programs aimed at developing drought-tolerant crops (Comas et al., 2013; Kim et al., 2020).

## Conclusion

The study identified significant differences in morphological and phytochemical traits among chickpea genotypes under drought stress. Genotypes MCC552 and MCC696 demonstrated superior drought tolerance, with higher PWUE, root length, and antioxidant enzyme activity. MCC552 showed improved root characteristics, while MCC696 excelled in physiological traits. Drought stress generally reduced shoot dry weight and leaf area but increased root depth and proline content. Notably, MCC552 displayed increased root diameter under stress. Photosynthetic rate and mesophyll conductance emerged as key factors influencing PWUE. Antioxidant enzymes (CAT, POD, GPX) showed increased activity with prolonged stress, especially in drought-tolerant genotypes. The consistent yield performance of MCC552 and MCC696 under both stress and non-stress conditions in field experiments (data not shown) further supports their potential as high-value candidates for drought tolerance breeding programs. These findings offer crucial insights for selecting and breeding drought-resistant chickpea varieties, enhancing crop yield under water-limited conditions.

## Data Availability

The data supporting this study’s findings are available from the corresponding author upon reasonable request.
