# Supplementary figures and images for: Evaluation of drought-tolerant chickpea genotypes (Cicer arietinum L.) using morphophysiological and phytochemical traits
Source: Front Plant Sci. 2025 Apr 9;16:1529177. doi: 10.3389/fpls.2025.1529177 (PMC12014700; doi:10.3389/fpls.2025.1529177)

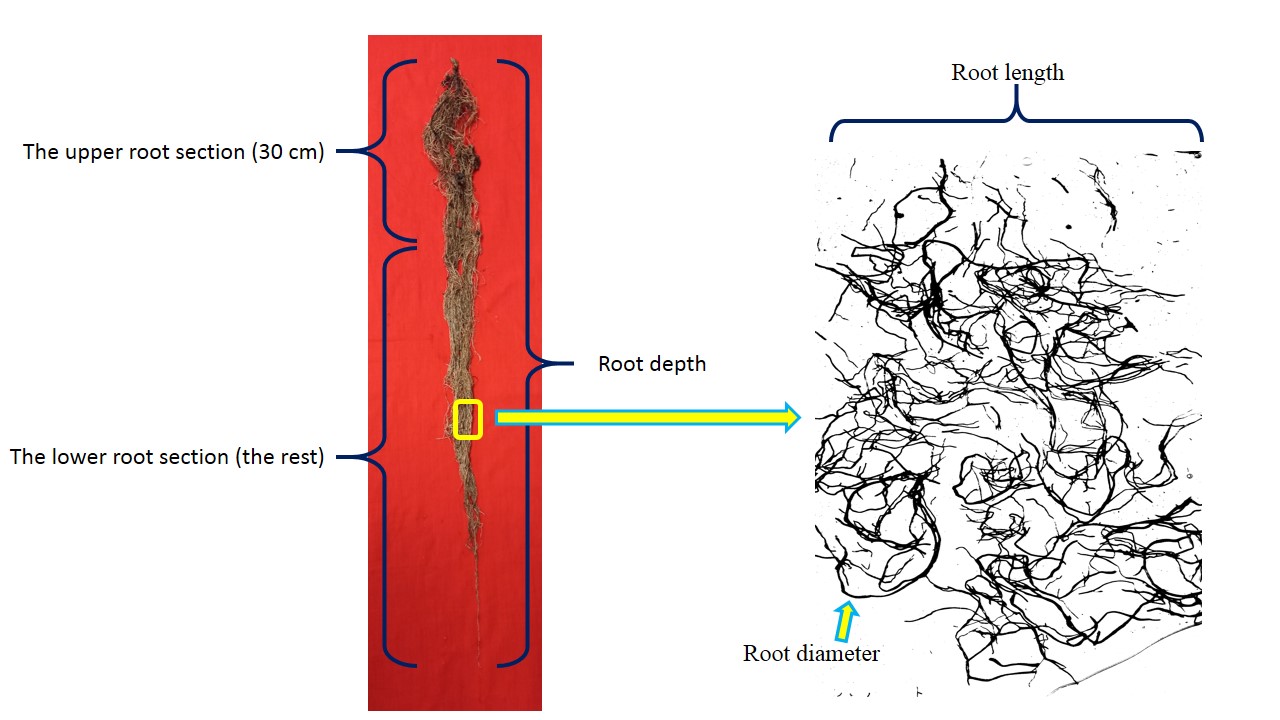

Supplement: Supplementary file 1 [file Image1.jpeg]
